# Is adiposopathy (sick fat) an endocrine disease?

**DOI:** 10.1111/j.1742-1241.2008.01848.x

**Published:** 2008-10

**Authors:** H E Bays, J M González-Campoy, R R Henry, D A Bergman, A E Kitabchi, A B Schorr, H W Rodbard, The Adiposopathy Working Group

**Affiliations:** 1 L-MARC Research CenterLouisville, KY, USA; 2 Minnesota Center for Obesity, Metabolism and Endocrinology (MNCOME)Eagan, MN, USA; 3 University of CaliforniaSan Diego, CA, USA; 4 San Diego VA Healthcare SystemSan Diego, CA, USA; 5 Department of Medicine, Division of Endocrinology Metabolism, and Metabolic Bone Diseases, Mount Sinai School of MedicineNew York, NY, USA; 6 The University of Tennessee Health Science CenterMemphis, TN, USA; 7 Subsection Head-Endocrinology, Saint Mary Medical CenterLanghorne, PA, USA; 8 Endocrine and Metabolic Consultants, RockvilleMD, USA

## Abstract

**Objective:**

To review current consensus and controversy regarding whether obesity is a ‘disease’, examine the pathogenic potential of adipose tissue to promote metabolic disease and explore the merits of ‘adiposopathy’ and ‘sick fat’ as scientifically and clinically useful terms in defining when excessive body fat may represent a ‘disease’.

**Methods:**

A group of clinicians and researchers, all with a background in endocrinology, assembled to evaluate the medical literature, as it pertains to the pathologic and pathogenic potential of adipose tissue, with an emphasis on metabolic diseases that are often promoted by excessive body weight.

**Results:**

The data support pathogenic adipose tissue as a disease. Challenges exist to convince many clinicians, patients, healthcare entities and the public that excessive body fat is often no less a ‘disease’ than the pathophysiological consequences related to anatomical abnormalities of other body tissues. ‘Adiposopathy’ has the potential to scientifically define adipose tissue anatomic and physiologic abnormalities, and their adverse consequences to patient health. Adiposopathy acknowledges that when positive caloric balance leads to adipocyte hypertrophy and visceral adiposity, then this may lead to pathogenic adipose tissue metabolic and immune responses that promote metabolic disease. From a patient perspective, explaining how excessive caloric intake might cause fat to become ‘sick’ also helps provide a rationale for patients to avoid weight gain. Adiposopathy also better justifies recommendations of weight loss as an effective therapeutic modality to improve metabolic disease in overweight and obese patients.

**Conclusion:**

Adiposopathy (sick fat) is an endocrine disease.

**What's known**

Excessive adipose tissue is generally accepted as a “cause” of clinical pathology related to its mass effects, including various cardiovascular, neurologic, pulmonary, musculoskeletal, dermatologic, gastrointestinal, genitourinary, renal, and psychological diseases.

**What's new**

It is less recognized, and sometimes disputed, that adipocyte hypertrophy and visceral adiposity may contribute (“cause”) metabolic diseases such as type 2 diabetes mellitus, hypertension, and dyslipidemia. Adiposopathy and “sick fat” are scientific and clinical terms, respectively, that help define when excessive body fat is a metabolic disease.

## Introduction

Obesity is an epidemic (1). An increase in body fat in many individuals and populations directly increases the risk of metabolic diseases such as type 2 diabetes mellitus (T2DM), hypertension and dyslipidaemia (2). These are the most common metabolic diseases encountered in endocrine practice, and might also be considered epidemics. However, obesity itself is not yet universally recognised as a disease (3). A sole focus on body mass index (BMI) in attempting to define obesity as a disease is not adequate (4). A more rational approach is to evaluate excessive body fat for its pathogenic potential. This requires recognising that adipose tissue is an active endocrine and immune organ (5), and that pathological disruption of important adipose tissue metabolic processes is detrimental to patient health.

Anatomically, positive caloric balance may lead to adipocyte hypertrophy and visceral adipose tissue accumulation, which are well-known contributors to metabolic disease (3,6). Conversely, weight loss interventions often help correct adipocyte and adipose tissue endocrine and immune abnormalities in overweight patients. This may lead to improvement in multiple metabolic parameters (7), often representing an effective therapy towards treatment of metabolic diseases such as T2DM, hypertension and dyslipidaemia (8).

The failure to adequately recognise the physiologic importance of adipose tissue to metabolic health, both clinically and in the medical/endocrine literature, is significantly because of a failure of existing terminology to adequately describe the pathogenic potential of adipose tissue, and its contribution to metabolic disease. An organ is often considered ‘diseased’ if it undergoes anatomic abnormalities associated with physiological dysfunction that ultimately lead to unfavourable health consequences. ‘Adiposopathy’ (adipose-opathy) is a term used to describe the adverse anatomical and pathophysiologic consequences of pathogenic adipose tissue. From a patient standpoint, the term adiposopathy can be translated as representing ‘sick fat’ (9). These terms and this approach emphasise that adipose tissue has as much pathogenic potential to result in ill health as the pathologic dysfunction of other body tissues. Thus, ‘adiposopathy’ represents a ‘disease’ similar to other organopathies.

## Adiposopathy and metabolic disease

**Figure 1:**
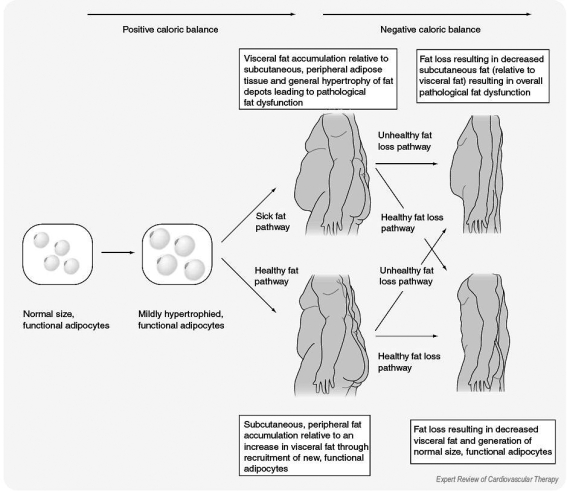
Anatomic manifestations of adiposopathy include adipocyte hypertrophy and visceral adiposity, which may lead to pathogenic metabolic and immune responses that promote metabolic disease (8). Positive caloric balance results in increased energy storage, which is initially manifested by mild adipocyte hypertrophy. This normally promotes paracrine signalling for adipogenesis (recruitment of new fat cells). Particularly when adipogenesis is impaired (3), continued positive caloric balance may worsen adipocyte hypertrophy, causing adipocytes to become dysfunctional and potentially pathogenic. Similarly, if excessive calories are stored in the visceral region, then this also is potentially pathogenic, and promotes metabolic disease. Excessive body fat may not be ‘healthy’ because of pathologic mass effects. However, accumulation of adipose tissue through adipocyte proliferation in the subcutaneous peripheral region may have less potential for promotion of metabolic disease, and may therefore be metabolically ‘healthier’. If during weight loss, subcutaneous peripheral adipose tissue is diminished and the proportion of visceral adipose tissue is increased, then this can also result in adiposopathy and promote metabolic disease, as is found with some cases of hypercortisolaemia and human immunodeficiency virus-associated lipodystrophy. Reproduced from *Expert Rev. Cardiovasc. Ther.* 4(6), 871–895 (2006) with permission of Expert Reviews Ltd

Adiposopathy is a disease characterised by pathogenic adipose tissue that is promoted by positive caloric balance and sedentary lifestyle in genetically and environmentally susceptible patients. Adiposopathy is anatomically manifested by adipocyte hypertrophy, visceral adiposity and/or ectopic fat deposition, which physiologically results in adverse endocrine and immune consequences leading to metabolic disease. During positive caloric balance, initial adipocyte hypertrophy optimally signals the recruitment, proliferation and differentiation of additional adipocytes in order to store energy (as fat) while maintaining normal adipose tissue functionality. However, if excessive fat cell enlargement occurs, such as when adipogenesis is impaired, then derangements of adipocyte (10) and adipose tissue metabolic and immune responses may lead to metabolic disease (Figure 1) (3).

**Figure 2:**
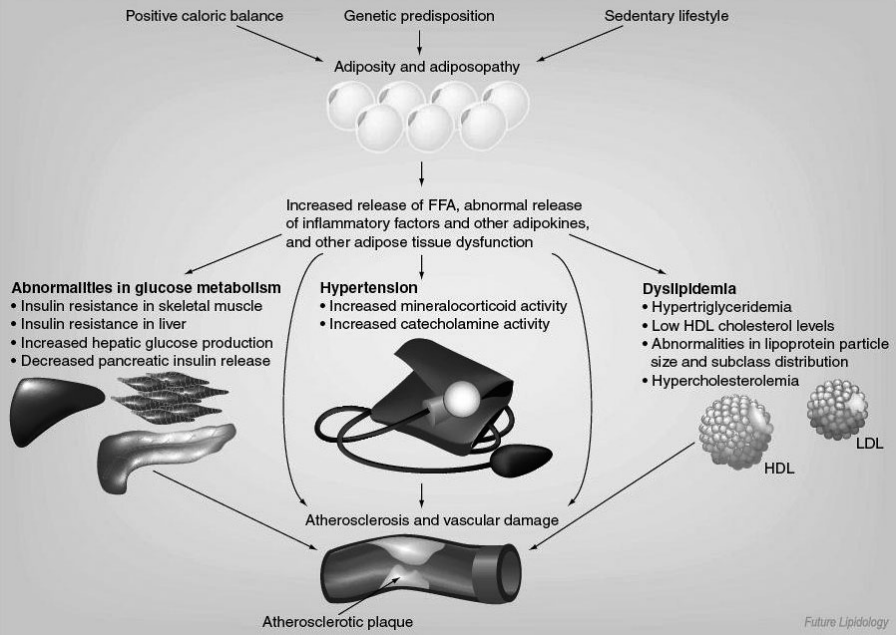
Adiposopathy is a disease that results in pathogenic metabolic and immune adipose tissue responses that promote metabolic disease (6). Age, gender, race, and genetic predisposition, and sedentary lifestyle are all examples of determinants as to how positive caloric balance may lead to adiposopathy. Pathogenic metabolic and immune responses associated with adiposopathy directly contribute to type 2 diabetes mellitus, hypertension, dyslipidaemia and potentially atherosclerosis. Reproduced from *Future Lipidol.* (2006) 1(4), 389–420 with permission of Future Medicine

Similarly, if positive caloric balance results in visceral adipose tissue accumulation, then this may also contribute to metabolic disease (Figure 1). Thus, it is not necessarily the increase in fat mass alone that leads to metabolic disease. Rather it is adipocyte hypertrophy and visceral adipose tissue adiposity that represents the pathologic anatomic abnormalities most likely to result in adverse metabolic consequences to patients (Figure 2).

The determination as to when positive caloric balance may lead to adipocyte hypertrophy and visceral adiposity is largely dependent upon underlying genetics and the surrounding metabolic environment (3). For example, obesity markedly increases the risk of T2DM among Pima Indians. Positive caloric balance often leads to hypertrophied adipocytes in this population, and the presence of anatomically larger adipocytes is a better predictor of the onset of T2DM, compared with obesity alone (11). Asian Indians often have an anatomically pathogenic adipose tissue presentation of increased adipocyte size and visceral adipose tissue accumulation, along with the pathophysiological metabolic and immune consequences of increased circulating free fatty acids, increased leptin, increased pro-inflammatory factors and decreased anti-inflammatory factors. As a result, they often have increased insulin resistance, T2DM and dyslipidaemia, as well as an increased risk of atherosclerotic coronary heart disease (CHD) (3). Hypercortisolaemia may reduce the size of adipocytes in peripheral, subcutaneous adipose tissue; but increase the relative, and possibly absolute accumulation of visceral adipose tissue. When coupled with a glucocorticoid-induced increase in appetite, hepatic gluconeogenesis and insulin resistance, this may all contribute to adverse metabolic and inflammatory adipose tissue responses that contribute to hyperglycaemia (3).

Another important determinant of the pathogenic potential of adipose tissue involves crosstalk and interactions with other body tissues. In fact, the degree by which pathogenic adipose tissue may ultimately result in metabolic disease is best considered a net pathologic partnership with limitations and/or dysfunction of other body organs. Disruption of biological signalling exchanges between adipose tissue and adjacent adipocytes, as well as impaired ‘crosstalk’ with the nervous system, immune system, skeletal muscle, cardiovascular system, liver, gastrointestinal system, adrenal cortex and thyroid, may all contribute to pathogenic endocrinologic and immune responses that contribute to metabolic disease (3). Additionally, the degree to which adipose tissue may contribute to metabolic disease is largely dependent upon the functionality of other non-adipose body organs. In one illustrative example, if adiposopathy results in the net release of excessive free fatty acids, then patients who have limitations in their ability to oxidise intramuscular fat or intrahepatic free fatty acids may be particularly predisposed to accumulate and store excessive triglycerides (3,12). This ectopic fat deposition may prove to be ‘lipotoxic’ to these organs and contribute to diabetes mellitus and dyslipidaemia (13). In summary, fat weight gain leading to metabolic disease is most dependent upon how fat is stored (adipocyte hypertrophy vs. adipocyte proliferation), where the fat is stored (visceral vs. other fat depots), and adipose tissue signalling and interactions with other body organs.

Metabolic diseases most associated with adiposopathy include T2DM, hypertension and dyslipidaemia; but may also include the metabolic syndrome, hyperandrogenemia in women and hypoandrogenemia/hyperestrogenemia in men (6). Adiposopathy may also directly contribute to atherosclerosis (6). The underlying manner and mechanisms whereby adiposopathy contributes to these metabolic diseases has been described in detailed elsewhere (3,6–8). But in general, these pathogenic mechanisms include impaired adipogenesis, visceral adiposity, increased net release of free fatty acids, deranged adipose tissue endocrine and inflammatory responses, and impaired crosstalk and/or impaired interactions with other body tissues (Table 1).

**Table 1:** Adiposopathy as a cause of metabolic disease: mechanistic supporting references (3,6–8)

	Type 2 diabetes mellitus	Hypertension	Dyslipidaemia
Impaired adipogenesis	(14–16)	(17,18)	(19)
Adipocyte hypertrophy	(13,20,21)	(22–24)	(21)
Visceral adiposity	(4,25,26)	(27,28)	(4,25)
Increased release of free fatty acids	(13,29)	(30,31)	(32)
Endocrinopathies	(33,34)	(33,35)	(33)
Inflammation	(36–38)	(39,40)	(41)
Impaired ‘crosstalk’ or impaired interactions with other body tissues	(42,43)	(44,45)	(46,47)

Positive caloric balance and sedentary lifestyle in genetically and environmentally susceptible patients leads to adipocyte hypertrophy (sometimes promoted by impaired adipogenesis), visceral adiposity and/or ectopic fat deposition. These anatomic abnormalities often result in pathophysiologic, adverse endocrine and immune consequences that lead to metabolic disease. Fat weight gain often results in pathologic adipose tissue dysfunction, accounting for the onset or worsening of type 2 diabetes mellitus, hypertension, dyslipidaemia and other metabolic disorders, which are the most common medical illnesses encountered in medical practice.

## Responses to challenges and claims regarding adiposopathy as a ‘disease’

Since 2006, through meetings, teleconferences and emails, the authors have discussed and documented various challenges and claims regarding the premise that adiposopathy is a reasonable term to describe pathogenic adipose tissue as a disease. ‘Real life’ specific challenges and claims listed below are derived from journal reviewer comments of publications previously published by the authors (3,7), and spirited correspondences and conversations with colleagues. In general, it is the experience of the authors that much disagreement exists regarding the most basic assertion that pathogenic adipose tissue directly contributes to metabolic disease. It is of interest that while many colleagues engaged by the authors have expressed the opinion that many of these premises are ‘self-evident,’ just as many have an entirely opposing opinion that these same premises have ‘yet to be proven.’ The following are examples of these challenges/claims, with the authors’ responses:

### Challenge 1: Metabolic activity of adipose tissue

**Claim**: Excessive fat mass does not have the potential to be a ‘disease’ because adipose tissue is generally an inactive metabolic organ with clinically insignificant endocrinologic and immunologic function.

**Response**: Adipose tissue is an active endocrine organ (3,33). Adipose tissue is an active immune organ (48). During times of positive caloric balance, and in patients who are genetically and/or environmentally predisposed, adipocyte hypertrophy and visceral adiposity may result in metabolic and immune abnormalities that directly contribute to metabolic disease (Table 1) (3,6).

### Challenge 2: Consistency in adipose tissue's pathogenic potential

**Claim:** Excessive adipose tissue does not have the potential to be a disease because obesity does not result in adverse metabolic consequences to all patients.

**Response:** Excessive adipose tissue may not be pathogenic in all patients. While many cases of obesity are associated with a significantly increased risk of various cardiovascular diseases (CVD) and cancer, and while combined overweight and obese patients are at risk for increased mortality from T2DM and kidney disease, many mildly to moderately overweight patients may have lower mortality risk from non-cancerous, non-CVD causes (49). Some studies even suggest that being overweight or obese provides ‘protection’ against atherosclerotic CHD (50). Overall, this suggests that functional adipose tissue, even when excessive, may be metabolically beneficial in some cases. This has sometimes described as representing an ‘obesity paradox’ (50). This paradox is largely resolved with an understanding that it is when adipose tissue is pathogenic that it then contributes to metabolic derangements. Adiposopathy also resolves another paradox wherein adding more functional adipocytes is employed as a therapeutic strategy to improve metabolic diseases that may be caused by excessive fat weight gain (Table 2). Peroxisome proliferator-activated receptor gamma agents increase the recruitment and proliferation of preadipocytes, and improve multiple metabolic parameters. This helps account for their efficacy in reducing glucose levels, and in some cases, improving lipid levels (3,7,13).

**Table 2:** Examples of treatments for adiposopathy and their effects upon adipose tissue factors that may contribute to metabolic disease (7)

Intervention	May affect glucose metabolism, blood pressure and lipid metabolism				May affect glucose metabolism	May affect blood pressure	May affect lipid metabolism	
	Visceral adipose tissue	Free fatty acids	Leptin	Adiponectin	Tumour necrosis factor-α	Renin-angiotensin-aldosterone enzymes	Androgens	Oestrogens
Nutrition and physical activity	↓	↓	↓	↑	↓	↓	↓ (women), ↑ (men)	↓/– (men)
PPAR-γ agonists(pioglitazone, rosiglitazone)	↓/–	↓	↓/–	↑	↓	–	↓	↓/– (men)
Orlistat	↓	↓	↓	↑	↓	?	↓ (women)	?
Sibutramine	↓	↓	↓	↑/–	?	?	↓ (women)	?
Cannabinoid receptor antagonists*	↓	↓	↓	↑	↓	?	?	?

Adipocyte hypertrophy and visceral adiposity result in multiple metabolic derangements that may promote metabolic disease. Existing therapies that treat adiposopathy (pathogenic adipose tissue), result in improvement in multiple adipose tissue metabolic parameters. This helps account for why adiposopathy treatments improve type 2 diabetes mellitus, hypertension and dyslipidemia (8).

* Not currently available in USA. ↑ = increased; ↓ = decreased; ? = unknown; – = neutral effect. PPAR-γ, peroxisome proliferator-activated receptor-γ.

### Challenge 3: Clinical importance of anatomic abnormalities of adipose tissue

**Claim:** Adiposopathy does not have the potential to be a useful scientific term because ‘disease’ is usually characterised by anatomical abnormalities of a body organ that leads to pathologic dysfunction, which in turn results in adverse clinical consequences to patients.

**Response:** During positive caloric balance, adverse metabolic consequences can be mitigated if the storage of excess energy is achieved through recruitment and proliferation of smaller, more functional adipocytes in peripheral, subcutaneous depots. Conversely, an increase in adipocyte size and an increase in visceral adipose tissue accumulation are both associated with metabolic abnormalities (51). If adipogenesis becomes impaired (either through genetic predisposition or environmental circumstances), then excessive adipocyte hypertrophy may occur in order to continue to store excess energy. It has been known since the 1970s that adipocyte hypertrophy results in pathogenic abnormalities that may lead to metabolic disease (10). It has been known since the 1940s that excessive visceral fat accumulation contributes to metabolic disease (52). Finally, another anatomic abnormality often associated with adipose tissue dysfunction is the deposition of ectopic fat, which may be ‘lipotoxic’ to other body organs, and again, lead to metabolic disease (13). Anatomically, adiposopathy is characterised by adipocyte hypertrophy, visceral adipose tissue accumulation and ectopic fat deposition. Thus, just as with other organ diseases, adipocyte and adipose tissue anatomic abnormalities are central to its pathogenic potential.

### Challenge 4: Differing inherent adipose tissue physiology based upon location

**Claim:** Adiposopathy does not have the potential to be a useful scientific term because not all adipose tissue is in the same location, and organs cannot become ‘diseased’ if they are in varying locations.

**Response:** Skeletal, smooth and/or cardiac muscle are examples of different muscle types located in different regions of the body. Each of these types of muscle has different physiologies, and different pathogenic potentials. Myocyte and muscle organ anatomic abnormalities may lead to disrupted physiology and adverse clinical signs and symptoms to patients. Muscle cell hypertrophy is an anatomic abnormality found in with some types of muscular dystrophies, and is a type of pathogenic “myopathy”. Similarly, adipose tissue is located in different locations, such as visceral, subcutaneous and perivascular regions. Not unlike muscle, different types of adipose tissue are widely distributed throughout the body, and have different physiologies and different pathogenic potentials, depending upon the depot. Thus, the anatomic abnormalities associated with adipocyte and adipose tissue, as described by the term ‘adiposopathy’, are potentially as much a ‘disease’ as with the ‘opathies’ of other body organs.

### Challenge 5: Differing pathogenic potential of adipose tissue depots

**Claim:** Adiposopathy does not have the potential to be a useful scientific term because it implies that different adipose tissues locations have the potential to contribute to metabolic ill health, when it is only visceral adiposity that is ‘pathogenic.’ Subcutaneous adipose tissue accumulation prevents metabolic disease, and is ‘protective’.

**Response:** Different fat depots inherently have different types and different degrees of metabolic activities (3,53). Accumulation and hypertrophy of visceral adipose tissue is most associated with an increased risk of metabolic disease (6). But the pathogenic potential of adipose tissue is not limited to visceral fat. Pericardial, perimuscular, perivascular, orbital and paraosseal adipose tissue are examples of periorgan adipose tissues that also may have pathogenic potential (54,55). Pericardial and perivascular adipose tissue may be pathogenic, through an ‘outside to inside’ model of atherosclerosis that directly promotes CHD and peripheral vascular disease (55–58). Although often considered ‘protective’, even subcutaneous adipose tissue can be pathogenic. For example, subcutaneous adipocyte hypertrophy located in the abdominal region may worsen metabolic disease (3,11,59).

Hypertrophy of peripheral subcutaneous adipose tissue may be pathogenic in other ways as well. Subcutaneous adipose tissue is the major source of circulating leptin (60). Leptin's secretion is associated more with adipocyte hypertrophy than with adipocyte hyperplasia (61). Hyperleptinaemia contributes to hypertension (62,63). Thus, excessive body fat storage through subcutaneous adipocyte hypertrophy may be pathogenic by promoting leptin-induced high blood pressure, although other adipose tissue mechanisms are likely involved as well (3,64). Another manner in which subcutaneous adipose tissue has pathogenic potential is in regard to free fatty acid release. The majority of postabsorptive systemic free fatty acids are derived from subcutaneous adipose tissue (65,66). These fatty acids may be ‘lipotoxic’ to muscle, pancreas and vasculature (65). Finally, it has even been suggested that the pathogenic potential of subcutaneous adipose tissue may sometimes be triggered by the pathogenic effects of visceral adipose tissue (65). Thus, the pathogenic potential of adipose tissue is not limited to visceral accumulation. The term ‘adiposopathy’ acknowledges this diversity in adipose tissue's pathogenic potential.

### Challenge 6: Contribution of body organs, other than adipose tissue, to metabolic disease

**Claim:** Adiposopathy does not have the potential to be a disease because the metabolic diseases often associated with obesity may significantly be due to the dysfunction of other body organs.

**Response:** The degree by which metabolic and immune derangements of pathogenic adipose tissue results in metabolic disease is dependent upon many factors such as the level of physical activity, genetic predisposition, age and environmental influences (e.g. drug therapies, dysfunction of other body organs, toxins, etc.). The response of other body organs to these derangements determines if adipocyte hypertrophy and visceral adiposity may ultimately result in clinical, metabolic disease. The pathogenic potential of adipose tissue is best viewed as a pathologic partnership with inherited or acquired limitations in ‘crosstalk’ and/or impairments of other body organs.

This type of relationship is neither novel nor unique to adiposopathy. Hyperglycaemia does not always result in acute or chronic diabetes complications; high blood pressure does not always result in cardiovascular or renal disease; and hypercholesterolaemia does not always result in clinical manifestations of atherosclerosis. Nonetheless, few would argue that these metabolic abnormalities are not ‘diseases’, or that somehow, the variabilities in clinical outcomes negates the need for their diagnosis and treatment (6). Instead, recommendations regarding diagnosis and treatment often target those at highest risk for adverse clinical outcomes as being the patients in most need of aggressive management. Similarly, pathogenic adipose tissue may not always result in clinical disease in all patients. Some patients may have sufficient inherent organ functionality (such as liver, muscle and pancreas) that allows for a heightened capacity to metabolise excessive free fatty acids and appropriately manage potential pathogenic metabolic and net pro-inflammatory adipocyte and adipose tissue responses. Conversely, other patients may have inherent [or acquired (67)] metabolic impairments of muscle (12,47,68–70), liver (71) and/or pancreas (72), such that they may be more susceptible to the adverse clinical consequences of pathogenic adipose tissue. Thus, just as with diabetes mellitus, hypertension and dyslipidaemia, pathogenic adipose tissue is a disease process that results in adverse clinical manifestations when in partnership with other concomitant facilitating factors. In the case of pathogenic adipose tissue, not only do adipocyte hypertrophy, visceral adiposity and ectopic fat deposition potentially contribute to the dysfunction of end organs such as liver, muscle and pancreas, but the inherited and/or acquired limitations or dysfunctions of these same end organs may exacerbate adiposopathy's potential to result in clinical metabolic disease.

### Challenge 7: Adipose tissue and personal behaviour

**Claim:** Adiposopathy cannot accurately be characterised as a ‘disease’ because excessive adipose tissue leading to metabolic disease is often the result of unfavourable personal behaviour whose effective therapy is unlikely to be achieved through medical science.

**Response:** Syphilis is a disease that periodically arises as an ‘epidemic’ in impoverished areas with lack of medical access, especially in patients with lower educational background who exhibit unfavourable personal behaviour (73). Syphilis was one of the leading causes of hopeless morbidity and mortality in the beginning of the 20th century. Initially, the cause was unknown and no diagnostic procedures or treatment existed. Subsequently, the cause was found, diagnostic criteria were established, and after ∼300 failures with other arsenical compounds, salvarsan was found to an effective treatment (74). This therapy has since been replaced by the even more effective treatment of penicillin.

Currently, many have little hope that an effective treatment for the epidemic of obesity is imminent. Diagnostic criteria for adiposopathy have yet to be established. It is unknowable if future therapies will be developed to treat pathogenic adipose tissue, with the same degree of efficacy as insulin in patients with diabetes mellitus, diuretics for hypertension and statins for hypercholesterolaemia (75). As with other metabolic diseases, combination therapies of agents with complementary mechanisms of actions may eventually be required in order to achieve optimal therapeutic goals (75). Additionally, public health factors must be overcome (73) in order to effectively treat adiposopathy. Such initiatives include better communication between healthcare providers and their patients, improved education about the pathogenic potential of adipose tissue (sick fat), implementation of more effective programmes to promote these public health initiatives, and personal adoption of more favourable nutritional and lifestyle habits.

From a pharmaceutical standpoint, few would argue that penicillin should be withheld from syphilis patients, irrespective of the degree to which unfavourable personal behaviour contributes to acquiring the disease. Similarly, while adiposopathy and its adverse metabolic consequences are often promoted by poor personal behaviour, it is nonetheless a disease that requires treatment. As has historically occurred whenever faced with seemingly hopeless epidemics, it is reasonable to believe that medical science will ultimately prevail. More effective therapies will be developed to effectively treat the epidemics of adiposopathy and its metabolic consequences (7,75).

### Challenge 8: Potential confusion in contrasting obesity with adiposopathy

**Claim:** Adiposopathy detracts from the essential message that excessive body fat is due to positive caloric balance, and potentially results in obesity which has adverse clinical consequences that are not restricted to metabolic and immune abnormalities. Excessive fat mass alone may cause adverse health consequences to patients.

**Response:** The authors concurs with prior clinical practice guidelines that call for the prevention and treatment of obesity beyond the metabolic consequences of adiposopathy, as excessive body fat mass alone can cause cardiovascular, neurologic, pulmonary, musculoskeletal, dermatologic, gastrointestinal, genitourinary, renal and psychological diseases (3,76,77). It is in these types of clinical presentations wherein the term ‘obesity’ alone might best be considered an underlying cause of adverse health consequences. However, it would be unreasonable to conclude that medical science, clinicians and patients are unable to comprehend the fundamental difference between the adverse physical health consequences associated with an increase in adipose tissue mass, and the adverse metabolic health consequences associated with adipose tissue dysfunction. The failure to recognise what may be two distinct pathologic entities may deny appropriate care to overweight and obese patients.

For example, many morbidly obese patients seek to treat their adiposity through surgical interventions, especially when they suffer from obesity-induced severe sleep apnoea, immobility, CVD and other such medical disorders (78). Although many of these surgical interventions may have a relatively high complication rate, especially in the presence of multiple comorbidities (79), they are often reimbursed through health insurance companies.

However, some patients with only mild increases in body weight may develop metabolic abnormalities that may place them at risk, or in fact cause diagnosable metabolic disease. This is one of the reasons why Asians require different BMI cut-off points in assessing their risk of metabolic disease (80). Because of the lack of established diagnostic criteria for pathogenic adipose tissue, access to care for these patients may be restricted because of a lack of third party payer coverage. To deny that adiposopathy can occur in even mildly overweight patients is to deny the most appropriate treatment to individual patients, and perhaps entire populations.

### Challenge 9: Adiposopathy is a novel term

**Claim:** Adiposopathy is an unnecessary term that does not aid clinicians in their assessment or treatment of patients, and ‘sick fat’ is unlikely to improve patient understanding of the relationship of excessive body weight to metabolic disease, as exists with more currently accepted terms such as the ‘metabolic syndrome’.

**Response:** The metabolic syndrome does not describe, nor does it attempt to describe a unified, underlying pathophysiologic process (81). In contrast, adiposopathy acknowledges that during positive caloric balance, adipose tissue may undergo pathogenic anatomical, metabolic and immune responses that lead to metabolic disease. From a practical standpoint, it would be most beneficial if clinicians could know which patients have pathogenic adipose tissue, and thus know which patients are most at risk for developing metabolic disease with weight gain. It would be of equal benefit to know which overweight patients would most likely have improvement in their metabolic disease with weight loss. Hence, it would be in patients’ best interest for scientific organisations and regulatory agencies establish diagnostic criteria for the diagnosis of adiposopathy (9). Once diagnostic criteria have been established, the next logical step would be creation of indications for treatment of adiposopathy, which in turn would treat important underlying causes of the most common endocrine diseases encountered in clinical practice (Table 2).

From a patient perspective, the term ‘sick fat’ is both accurate and perhaps clinically useful. A discussion as to how increasing body weight may cause their fat to become ‘sick,’ or how losing body weight may cause their fat to become more ‘healthy’, might be a more fruitful discussion than discussing the diagnostic components defining the ‘metabolic syndrome (82)’.

### Challenge 10: Adiposopathy is a ‘novel’ concept

**Claim:** Adiposopathy is not a potentially useful scientific term because it emphasises the profound metabolic and immune pathogenic potential of adipose tissue whose complexities will present an insurmountable educational challenge.

**Response:** Max Planck, founder of quantum physics, suggested in the mid-1900s: ‘A new scientific truth does not triumph by convincing its opponents and making them see the light, but rather because its opponents eventually die, and a new generation grows up that is familiar with it’ (81). His intent was to describe his perception of the challenges associated with scientific concepts not yet accepted by the scientific establishment. Arthur Schopenhauer, a German philosopher (1788–1860) is credited with saying that: ‘All truth passes through three stages. First, it is ridiculed. Second, it is violently opposed. Third, it is accepted as being self-evident’. So, it may also be that even some in the scientific community may be resistant to the suggestion that pathogenic adipose tissue is a disease. But in contrast to the scientific establishment, it is simply a fact that clinicians often see patients who are markedly overweight, yet have no diagnosable metabolic disease. They also see other patients who, upon gaining only modest body fat, develop T2DM, hypertension and dyslipidaemia. For many of them, ‘adiposopathy’ may prove to be a welcomed term that better reflects their practical, clinical experience regarding overweight patients. ‘Sick fat’ is a term that might better assist them in educating their patients.

## Conclusion

Adiposopathy is an endocrine disease.
